# Oocyte aging is controlled by mitogen‐activated protein kinase signaling

**DOI:** 10.1111/acel.13386

**Published:** 2021-06-01

**Authors:** Hanna Achache, Roni Falk, Noam Lerner, Tsevi Beatus, Yonatan B. Tzur

**Affiliations:** ^1^ Department of Genetics Institute of Life Sciences The Hebrew University of Jerusalem Jerusalem Israel; ^2^ Department of Neurobiology The Institute of Life Science The Hebrew University of Jerusalem Jerusalem Israel; ^3^ The Alexander Grass Center for Bioengineering The Rachel and Selim Benin School of Computer Science and Engineering The Hebrew University of Jerusalem Jerusalem Israel

**Keywords:** aging, *C. elegans*, fertility, MAPK, meiosis, oocyte, oogenesis

## Abstract

Oogenesis is one of the first processes to fail during aging. In women, most oocytes cannot successfully complete meiotic divisions already during the fourth decade of life. Studies of the nematode *Caenorhabditis elegans* have uncovered conserved genetic pathways that control lifespan, but our knowledge regarding reproductive aging in worms and humans is limited. Specifically, little is known about germline internal signals that dictate the oogonial biological clock. Here, we report a thorough characterization of the changes in the worm germline during aging. We found that shortly after ovulation halts, germline proliferation declines, while apoptosis continues, leading to a gradual reduction in germ cell numbers. In late aging stages, we observed that meiotic progression is disturbed and crossover designation and DNA double‐strand break repair decrease. In addition, we detected a decline in the quality of mature oocytes during aging, as reflected by decreasing size and elongation of interhomolog distance, a phenotype also observed in human oocytes. Many of these altered processes were previously attributed to MAPK signaling variations in young worms. In support of this, we observed changes in activation dynamics of MPK‐1 during aging. We therefore tested the hypothesis that MAPK controls oocyte quality in aged worms using both genetic and pharmacological tools. We found that in mutants with high levels of activated MPK‐1, oocyte quality deteriorates more rapidly than in wild‐type worms, whereas reduction of MPK‐1 levels enhances quality. Thus, our data suggest that MAPK signaling controls germline aging and could be used to attenuate the rate of oogenesis quality decline.

## Graphical abstract

In *C. elegans* germline, aging leads to several changes, including crossover designation, apoptosis, and the quality of the mature oocytes. All of these were previously shown to be controlled by MAPK signaling. We show that arrested oocyte's quality is lower when MAPK is overactivated, yet it can be improved by lowering MAPK via genetic or pharmacological tools.

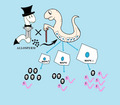

## 1 INTRODUCTION

Aging leads to a gradual decline and failure of physiological processes. One of the first processes to fail during metazoan aging is oogenesis (Andux & Ellis, 2008; Greenblatt et al., 2019; Gruhn et al. 2019; Luo et al., 2010; Nagaoka et al., 2012; Webster & Schuh, 2017). In women, oocytes enter meiosis during maternal embryogenesis, arrest at the end of meiotic prophase I, and remain quiescent for decades. Over time, the quality and quantity of these oocytes decreases, concurrently with reduced fertility and increased occurrence of aneuploidy (Bentov et al., 2011; Duncan et al., 2012; Eichenlaub‐Ritter et al., 2011; Lord & Aitken, 2013; te Velde & Pearson, 2002). Yet, our knowledge of the genetic and molecular mechanisms that govern this aging process is currently limited.

Studies in the nematode *Caenorhabditis elegans* have played pivotal roles in our understanding of the genetic contribution to longevity and aging. The *C*. *elegans* system has the advantages of short lifespan (2–3 weeks), simple genetic setup, and the evolutionary conservation of the longevity pathways (Kenyon, 2010; Luo et al., 2010; Wilkinson et al., 2012). Several inherent properties make *C*. *elegans* highly suitable for the study of germline aging (Andux & Ellis, 2008; Hughes et al., 2007; Luo et al., 2009). First, oogenesis is continuous and the nuclei in the adult gonad are ordered in a spatio‐temporal manner from the germ line stem cells to the mature oocyte (Crittenden et al., 1994; Hillers et al., 2017; Lui & Colaiacovo, 2013; Pazdernik & Schedl, 2013). Second, in hermaphrodites, ovulation is uninterrupted as long as self‐sperm is available. Once sperm is depleted, oocytes arrest at the end of meiotic prophase I. The hermaphrodite worm remains fertile for several more days and can resume ovulation and fertilization upon mating with males as a response to the introduction of allosperm into the uterus (Andux & Ellis, 2008; Chasnov, 2013; Hodgkin, 1983; Hughes et al., 2007; Kocsisova et al., 2019; Mendenhall et al., 2011; Pickett et al., 2013). Therefore, unlike human oocytes, worm oocytes are continuously produced because the germ cell population is proliferative (Crittenden & Kimble, 2008; Crittenden et al., 2006). Finally, both human and *C*. *elegans* females reproduce for about one‐third of their lifespan (Hughes et al., 2007) and thus undergo reproductive aging on proportional time scales.

Building upon these properties, several previous works described different aspects of oogenesis at certain phases of germline aging in *C*. *elegans* (Andux & Ellis, 2008; Bohnert & Kenyon, 2017; Hughes et al., 2007; Luo et al., 2009, 2010; Templeman & Murphy, 2018; Wang et al., 2014; Ye & Bhalla, 2011). These works showed that aging oocytes undergo gross morphological and functional changes. Morphological changes include the presence of small stacked oocytes and endomitotic nuclei in the proximal gonad (de la Guardia et al., 2016; Kocsisova et al., 2019). Functional defects involve reduced embryo hatching and stress resistance, decreased oocyte fertilizability, altered crossover distribution, and high incidence of males (Lim et al., 2008; Luo et al., 2010; Nagaoka et al., 2012; Perez et al., 2017). *C*. *elegans* naturally exists as two sexes: XX hermaphrodites and XO males. X chromosome ploidy determines sex in *C*. *elegans* (Rose & Baillie, 1979). Therefore, a high incidence of males is indicative of increased aneuploidy as a result of meiotic mis‐segregation of the X chromosome. Age‐related increase in progeny aneuploidy is also observed in humans (Nagaoka et al., 2012). Mutations in several genetic pathways extend the fertility term (Hughes et al., 2011; Luo et al., 2010). Among these, mutations in genes encoding factors involved in the insulin/IGF‐1 signaling (IIS), the TGF‐β‐Sma/Mab, and the dietary restriction pathways extend the fertility period in worms from both self and allosperm (Reviewed in (Lopez‐Otin et al., 2013)). Nevertheless, to date no systematic work has analyzed the dynamics of major meiotic processes along all steps of germline aging. Moreover, the germline signals that lead to the specific oogonial changes during normal aging are still largely unknown.

Signals that control developmental processes are often also involved in aging (Blagosklonny & Hall, 2009; Gruber et al., 2016; Slack, 2017). We therefore hypothesized that some signaling pathways that are activated during oogenesis also influence germline and oocyte aging. The MAPK pathway controls oogenesis progression in *C*. *elegans* (Kim et al., 2013; Lee et al., 2007; Nadarajan et al., 2016). Several proteins promote or restrict the activation of its terminal kinase, MPK‐1, the worm homolog of ERK, and this, in turn, leads to multiple transcriptional and post‐transcriptional cellular changes that drive oogonial processes (Achache et al., 2019; Arur et al., 2011; Church et al., 1995; Kritikou et al., 2006; Lackner & Kim, 1998; Leacock & Reinke, 2006; Lee et al., 2007; Yin et al., 2016).

Here, we show that meiotic progression is altered and processes such as double‐strand break repair and crossover designation are reduced in the *C*. *elegans* germline during aging. These alterations occur concomitantly with a change in spatial activation of MPK‐1. During aging, oocyte quality was inversely correlated with and dependent on the level of MAPK activation. Furthermore, in mutants with high levels of activated MPK‐1, oocyte quality deteriorated more rapidly than in wild‐type worms, whereas reduction of MPK‐1 levels enhanced quality. We conclude that MAPK signaling in mature oocytes controls reproductive aging by influencing oocyte and germline quality.

## 2 RESULTS

### 2.1 Germline aging leads to a reduction in germ cell numbers and altered meiotic staging

Although *C*. *elegans* hermaphrodites make both oocytes and sperm, the differentiation of germ cells is sequential. At the third larval stage (L3), the worms start to produce sperm and then switch to oogenesis in the fourth larval stage (L4, reviewed in (Schedl, 1997)). Oocytes start to be fertilized at the adult stage, and hermaphrodites continue to ovulate until most of the self‐sperm is depleted, at which point the oocytes arrest and age (Kim et al., 2013; Templeman & Murphy, 2018)). To study germline aging, we chose to use the N2 wild‐type strain instead of feminized mutants as was done previously (e.g. (Andux & Ellis, 2008; Bohnert & Kenyon, 2017; de la Guardia et al., 2016; Hughes et al., 2007; Lim et al., 2008; Luo et al., 2010; Templeman & Murphy, 2018)). Our strategy ensured that the aging effects we detected were unrelated to any mutation. Previous work has shown that most self‐progeny of wild‐type worms are laid at the second day post‐L4 and that almost all the embryos are laid within three days (Hughes et al., 2007; Pickett et al., 2013; Wang et al., 2014). We verified this result under our experimental conditions (Figure S1). A negligible number of oocytes were laid after three days post‐L4 (under 0.8 on average per worm), and no viable embryo was laid after the fifth day (Figure S1). Therefore, we defined four points during the aging process: the onset of reproduction, the beginning of the arrest, the end of the reproductive term by male cross‐fertilization, and after the reproductive term (Hughes et al., 2007), which can also be described as young, mature, old, and menopausal. These stages are observed at one, four, eight, and ten days post‐L4, respectively.

**FIGURE 1:**
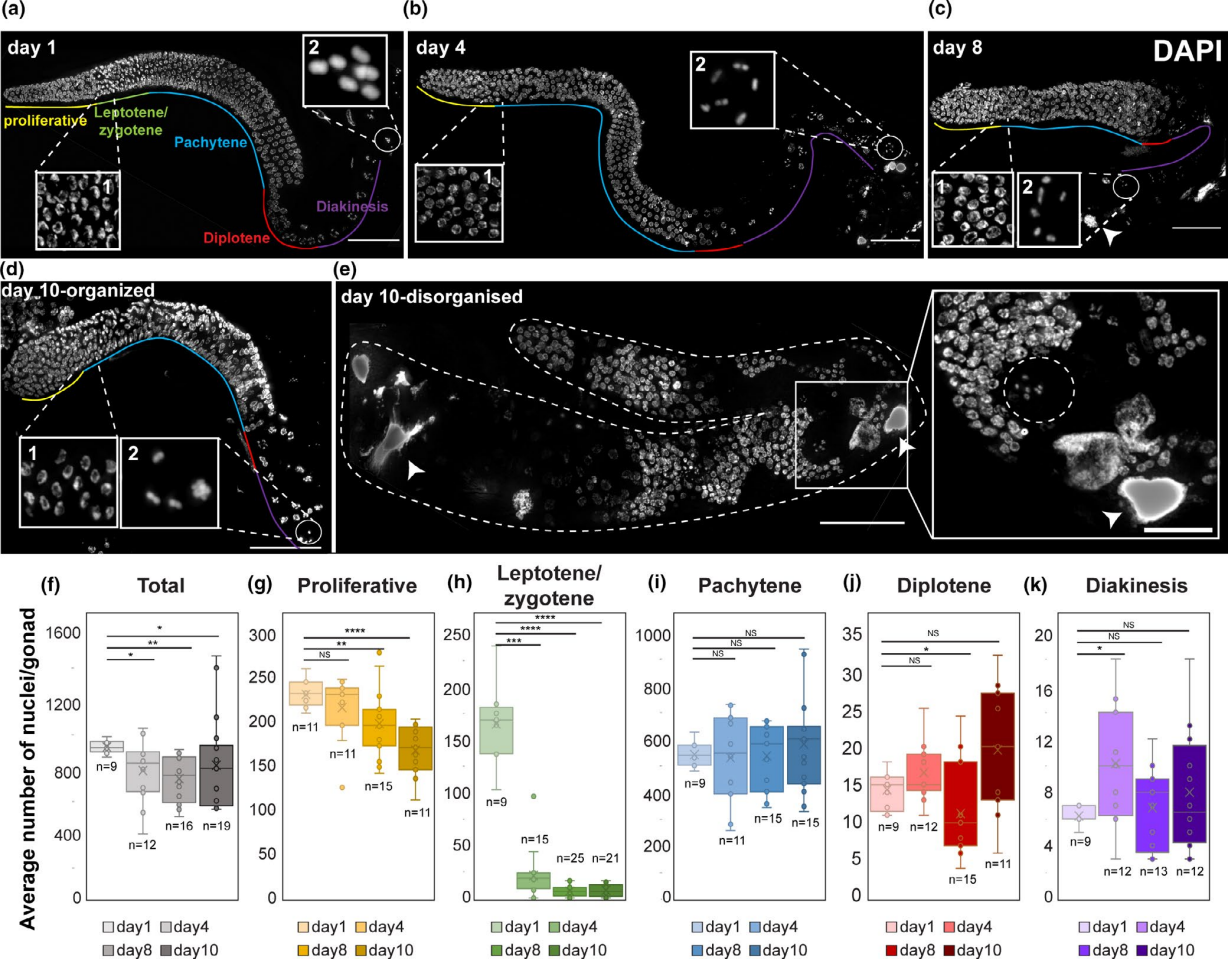
Aging leads to reduced number of germ cells. (a–e) Images of DAPI‐stained whole mount gonads from worms at the indicated ages. The different oogonial stages are marked. Insets show regions containing LZ nuclei (1) and mature oocytes (2). Two categories of day 10 gonads are shown: organized and disorganized. Arrowheads—endomitotic nuclei. Dashed circle in (e) indicates a nucleus with diakinesis morphology located distally. Scale bar = 50 µM. (f–k) Box plots depict the average number (*x*) and the individual measurements (circles) of (f) total nuclei in the gonads and (g–k) specific stages per gonad from worms at the indicated ages. Mann–Whitney *p* value: NS—not significant, *<0.05, **<0.01, ***<0.001, and ****<0.0001. *n* values are marked below each box

To find how aging affects germ cell number and developmental stages, we examined dissected gonads stained with DAPI. The *C*. *elegans* gonad is comprised of two U‐shaped arms with nuclei arranged in spatial‐developmental order. The proliferative zone is located at the distal end of each arm, and mature oocytes and the spermatheca are found at the proximal end. Nuclei in the proliferative zone undergo mitotic cell cycles to maintain a population of progenitors that enter meiosis in the leptotene/zygotene (LZ, transition) zone. From there, nuclei progress through pachytene, where recombination intermediates mature into crossovers within paired and synapsed homologs. Pachytene nuclei move into diplotene, and finally oocytes mature and cellularize in diakinesis, where six discrete bivalents can be visualized (Figure 1a inset 2). We counted the total number of nuclei in DAPI‐stained gonads and observed an overall decrease with age (Figure 1a‐f). We found high variability in the number of germ cells in day 10 worms. 16% (*n* = 3/19) of the gonads contained over 1000 nuclei (Figure 1e), while we also found gonads with as little as 550 nuclei. The overall reduction in the number of germ cells along aging could stem from either lower proliferation or accelerated apoptosis (see below). To test whether the reduction in germ cell numbers occurs in the same relative percentages in all meiotic stages or is it in specific stages, we quantified the numbers of nuclei at each meiotic stage (Figure 1g–k). The number of crescent‐shaped nuclei (LZ) quickly dropped and was reduced at the onset of oocyte arrest (day 1: 132±30, *n* = 9; day 4: 17±19, *n* = 15; day 8: 5±4, *n* = 25; day 10: 6±5, *n* = 21; Figure 1h). We also detected a decrease in the number of proliferative nuclei during aging (day 1: 230±15, *n* = 11; day 4: 215±36, *n* = 11; day 8: 197±38, *n* = 15; day 10: 167±29, *n *= 11; Figure 1g). Thus, germline aging and arrest lead to reductions in germ cell numbers, mostly due to a rapid drop in the number of mitotic and LZ nuclei. A change in the relative number of meiocytes has been previously linked to control of meiotic progression due to aberrant MPK‐1 activation (Achache et al., 2019; Hajnal & Berset, 2002; Lee et al., 2006; Lin & Reinke, 2008) (see below).

The spatial temporal order in the young adult gonad has been highly advantageous in meiotic studies in this model organism. This order was always present in day 1 and day 4 worms. However, starting at day 8 after the L4 stage, we noticed an increased number of gonads with altered morphology. In these gonads, we found a high number of nuclei and meiotic progression was disorganized, such as diakinetic‐like nuclei with large cytosolic volumes were observed along the middle of the gonad, while pachytene‐like nuclei were observed proximally (Figure 1e). We found 3.5% disorganized gonads at day 8 (*n* = 1/29 gonads) and 19% at day 10 (*n* = 5/27 gonads)). The heterogeneity of both organized and disorganized gonads within the same age‐matched population could be the result of different aging fates (see discussion). The mixture of meiotic stages in the disorganized gonads indicates a loss of meiotic progression control. A similar phenotype was reported previously in mutants of *kin*‐*18* (Yin et al., 2016), an activator of MAPK (see below). Unless otherwise mentioned, all further data regarding day 8 and 10 gonads relate to the majority of the population, containing gonads with organized nuclei (Figure 1c,d). Taken together, these analyses suggest that germline aging leads first to a reduction in the number of germ cells and in some gonads to misregulation of meiotic progression.

### 2.2 Germ cell proliferation declines with aging

**FIGURE 2:**
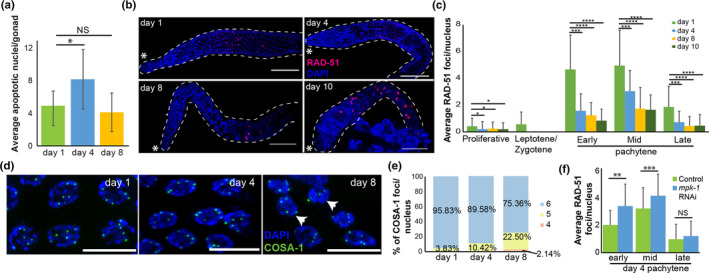
Changes in meiotic processes in aged germlines. (a) Average number of CED‐1::GFP apoptotic nuclei per gonad from worms at the indicated ages. *n* ≥ 7 gonads. Mann–Whitney *p* value: NS—not significant, *<0.05. (b) RAD‐51 (red) and DAPI (blue) staining of whole mount gonad arms at the indicated ages. Scale bar = 50 µM. Asterisk indicates the distal side of the gonad. (c) Average number of RAD‐51 foci per nucleus at the different oogonial stages. (d) COSA‐1::GFP (green) and DAPI (blue) staining of late pachytene nuclei at the indicated ages. Scale bar = 10 µM. (e) Histogram of the relative percentages of nuclei with four, five, and six COSA‐1 foci per late pachytene nucleus at the different ages. *n* = 200 nuclei (f) Average number of RAD‐51 foci per gonad nuclei at different pachytene stages in wild‐type day 4 gonads of control and *mpk*‐*1* RNAi worms. *n* ≥ 80 gonads. Mann–Whitney *p* value: NS—not significant, *<0.05, ***<0.001, and ****<0.0001

The number of nuclei in the gonad is tightly regulated by a dynamic balance between germ cell proliferation and removal by both oocyte ovulation and apoptotic cell death (Lettre & Hengartner, 2006). When sperm are depleted, ovulation ceases almost completely. The reduction in the number of germline nuclei may therefore be due to either an increase in apoptosis or a decrease in mitotically proliferating nuclei. To test the former, we used a strain stably expressing CED‐1::GFP, a fusion protein that is expressed in somatic sheath cells, which cluster around each apoptotic corpse during engulfment (Schumacher et al., 2005; Zhou et al., 2001). This approach is particularly useful for detecting early apoptotic stages. In agreement with a previous publication (de la Guardia et al., 2016), in young adult worms we found fewer apoptotic nuclei (4.6±2.1, *n* = 8) than in mature worms (8.2±3.6, *n* = 11), but the trend was reversed in old worms (4.1±2.4, *n* = 7, Figure 2a). We were unable to quantify apoptotic levels at day 10, since almost all the transgenic CED‐1::GFP worms died before reaching day 10. These results suggest that at the onset of aging, the apoptotic removal of meiocytes increases but then returns to young worm levels.

The increase in apoptosis was transient, whereas the reduction in germ cell numbers was continuous. To investigate whether proliferation also regulates the overall number of nuclei in the gonad, we monitored the number of germ cells in M phase at days 1, 4, 8, and 10 by staining for a mitosis‐specific marker phospho‐histone H3 (pH3) (Hans & Dimitrov, 2001). We detected a rapid decline in the number of pH3‐positive nuclei in the distal region at day 4, and at days 8 and 10, no nuclei were stained with pH3 (Figure S2). Taken together, these results suggest that aging leads to reduction in the number of germ cells due to both reduced proliferation and increased apoptosis.

### 2.3 Reduced levels of RAD‐51 foci in aging gonads

Apoptosis in the worm germline has been correlated with aberrations in DNA double‐strand break repair and synapsis (reviewed in (Gartner et al., 2008)). To test whether the increase in apoptosis is induced by altered dynamics of homologous recombination repair, we quantified the number of repair loci using RAD‐51 staining. RAD‐51 is a strand exchange protein that has been used extensively to study the DNA double‐strand break repair dynamics in the *C*. *elegans* gonad (Alpi et al., 2003; Bhalla & Dernburg, 2005; Colaiacovo et al., 1999; Hayashi et al., 2007; Mets & Meyer, 2009; Rinaldo et al., 2002; Yu et al., 2016). In the young adult worms, the levels of RAD‐51 rose following entry into meiotic prophase I and peaked in the early to mid‐pachytene stage (Figure 2b,c), as previously reported (Achache et al., 2019; Colaiacovo et al., 2003). Numbers of RAD‐51 foci were greatly reduced in nuclei at all the stages of meiotic prophase I in aged worms (Figure 2b,c). Indeed, at day 1, we found an average of 4.7±2.6 foci per nucleus in early pachytene, compared to 1.6±1.3, 1.3±1, and 0.9±0.9 at days 4, 8, and 10, respectively. Thus, after the halt in ovulation, the number of RAD‐51 foci drops. This drop in the number of RAD‐51 foci might be explained by a progression of the arrested nuclei beyond the removal of RAD‐51, together with a reduction in further induction of double‐strand breaks. Another option could be a defect in either the induction or the repair of the DNA double‐strand breaks. Interestingly, in gonads of days 8 and 10, we detected nuclei in which RAD‐51 staining filled the entire nucleoplasm (Figure 2b, 8±5, *n* = 11 gonads for both). This type of staining could be the result of fragmented DNA or misregulation of RAD‐51 expression. Other than those nuclei, the dynamics of RAD‐51 foci was similar between day 4 and later time points. Taken together, our results suggest that the increase in apoptosis observed on day 4 is unlikely to be the result of perturbations in the DNA repair mechanism.

### 2.4 Synaptonemal complex formation is unchanged during germline aging

During meiosis, the formation of a proteinaceous structure known as the synaptonemal complex (SC) stabilizes pairing interactions and promotes the completion of crossover recombination. SCs assemble along the lengths of the paired chromosomes to keep them closely associated and aligned (Colaiacovo et al., 2003; Couteau & Zetka, 2005; Hayashi et al., 2010; Schild‐Prufert et al., 2011). This zipper‐like structure is composed of lateral element proteins that are recruited to the chromosome axes and to central region proteins that localize between them and keep the homologs aligned (reviewed in (Zetka, 2009)). Failure to properly form the SC increases apoptosis levels in the gonad (Bohr et al., 2016). To test whether the increase in apoptosis levels on day 4 relative to day 1 is correlated with aberrant synapsis, we performed immunostaining using antibodies against SYP‐2, a central region protein (Colaiacovo et al., 2003), and HTP‐3, an axial component of the SC (Goodyer et al., 2008; MacQueen et al., 2002; Severson et al., 2009). In mid‐pachytene stages, these proteins co‐localize on the DAPI‐stained tracks in young adult worms (Figure S3), indicating proper formation of the SC. We observed similar patterns at all aging time points with no indications of loss or partial synapsis (Figure S3). Thus, at least at the level of this observation, synapsis is not altered with germline aging. Taken together, these results open the possibility that factors other than synapsis and repair of DNA double‐strand breaks contribute to the increased apoptosis observed on day 4 after L4 in worm gonads.

### 2.5 Germline aging leads to small reduction in crossover designation

Interhomolog crossover recombination is dependent on proper repair of DNA double‐strand breaks, and reduced crossovers have been suggested to play roles in aneuploidy in advanced aged mothers (reviewed in (Webster & Schuh, 2017)). To determine whether the reduction in RAD‐51 foci numbers with maternal age is accompanied by changes in crossover designation, we examined the loading of GFP‐tagged COSA‐1 onto meiotic chromosomes. COSA‐1 localizes to the single crossover site in each homolog pair in late prophase I (Yokoo et al., 2012) and is the earliest known marker for crossovers in *C*. *elegans*. At day 1, almost all nuclei (96%) in the last five rows of the pachytene stages of the young adult gonad showed six COSA‐1 foci corresponding to the six crossover sites per nucleus present in *C*. *elegans* (Figure 2d,e). Aging led to a gradual decrease in crossover designation. On day 4, 10.4% of the nuclei had five foci, and on day 8, 22.5% had only five foci (Figure 2d,e). Moreover, on day 8 after L4, 2% of the nuclei in late pachytene had only four COSA‐1::GFP foci (Figure 2d,e). Interestingly, a reduction in the number of COSA‐1 foci was observed in late prophase I stages in *kin*‐*18* mutants (Yin et al., 2016). Similar to the CED‐1::GFP worms, almost all the worms of COSA‐1::GFP transgenic strain died before day 10, and thus, we were unable to collect relevant data for that stage. These results suggest that aging leads to reduction in crossover recombination designation.

### 2.6 The distance between homologous chromosomes increases in old worms

In human, the percentage of meiotic chromosomal mis‐segregation exponentially increases with maternal age (Hassold & Hunt, 2001; Koehler et al., 2006). This has been attributed to the time oocytes are arrested, and, indeed, increased premature dissociation of chromosomes has been observed in oocytes in aging women (Lister et al., 2010; Subramanian & Bickel, 2008; Tsutsumi et al., 2014). Nevertheless, the magnitude of premature dissociations is lower than the aneuploidy rate, suggesting that other factors control the arrested oocyte quality and potential to complete the divisions (Nagaoka et al., 2012). The increased levels of oocytes with only five COSA‐1::GFP foci in old worms raise the possibility that aging leads to reduced crossovers, which in turn should lead to presence of univalent chromosomes in mature oocytes.

In young adult worms, the six bivalents of *C*. *elegans* are almost always detected as six separate DAPI‐stained bodies. When homologous chromosomes either do not undergo crossovers or separate before anaphase I, more than six bodies are expected. We did not observe an increase in extra bodies in aged oocytes (Figure 1a,e). In fact, we noticed an increase in the number of oocytes in which the bivalents were in very close proximity, and these chromosomes seemed connected at the resolution level of our microscopy system (12.5% of the mature oocytes contained 5 or less bivalents at day 10 vs. 3% at day 1, Figure 1a,d inset 2, *n* ≥ 32 gonads). This suggests that aging does not lead to nuclei with non‐crossover chromosomes; however, nuclei with non‐crossover chromosomes may be removed by apoptosis.

**FIGURE 3:**
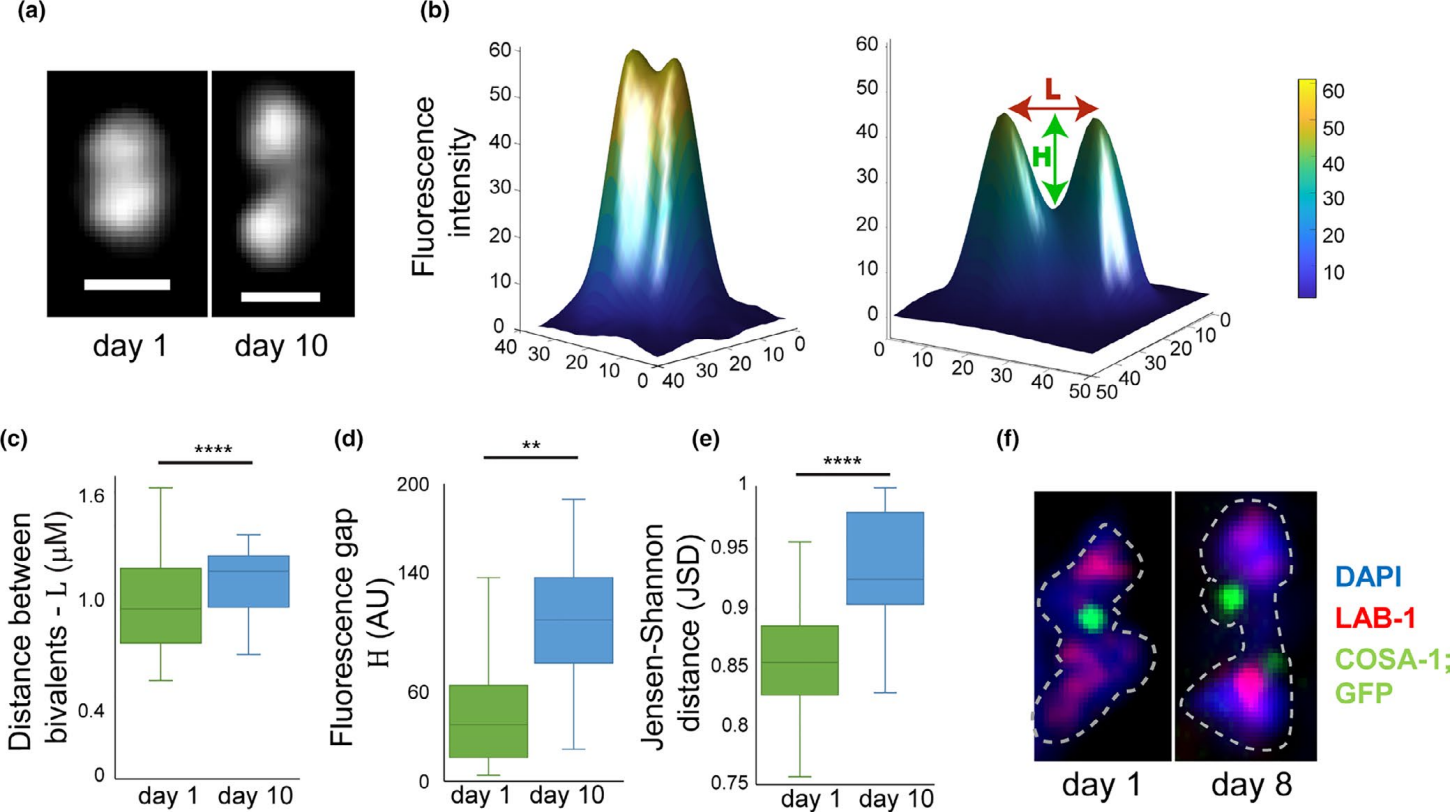
Homologs are located at greater distance from each other in old worms. (a) The z‐projected images of DAPI‐stained bivalents in mature oocytes at day 1 and day 10. Scale bar = 1 µM. (b) 2D chromatin density maps obtained by projecting the z‐stacks in panel (a) for day 1 and day 10 bivalents from mature oocytes. Chromatin density is indicated both by color and surface height. The 2D distance between the projected Gaussian centers is defined as *L*. The difference in fluorescence between the minimum value along *L* and the mean value of the two Gaussians centers is defined as *H*. (c) Box and whisker plot of the distance *L*. *n *= 20. (d) Box and whisker plot of the fluorescence difference *H*. *n* = 20. (e). Box and whisker plot of the Jensen–Shannon distance between the 3D Gaussian distributions of the two homologs in each pair. *n* = 20. (f) Images of bivalents from day 1 and day 8 mature oocytes stained for LAB‐1 (red), COSA‐1::GFP (green), and DAPI (blue). *n* = 32

Although homologs are attached during diakinesis, we hypothesize that this attachment weakens with age. This hypothesis predicts that homologs in aged oocytes are more spatially separated than in young oocytes, as previously observed in mouse and human (Gruhn et al., 2019; Zielinska et al. 2019). To test this hypothesis, we used a fluorescence microscope to capture the 3D chromatin density of the chromosomes in mature oocytes in both young and menopausal worms (Figure 3a). To identify individual chromosomes within each pair of homologs, we used an unbiased 3D Gaussian mixture model with two Gaussians that was fitted to the measured chromatin density. The spatial separation between the homologs was quantified by projecting all images in the z‐stack into a single 2D chromatin density map and calculating the 2D distance (*L*) between the projected centers of the two Gaussians (Figure 3b). This analysis showed that *L* is significantly longer in oocytes of menopausal vs. young worms (Figure 3c).

If this longer distance is caused by weakening of the sister chromatid cohesion, then less chromatin is expected to be present at the interface between the two homologs (known as the short arms). To test this prediction, we analyzed the fluorescence profile between the two peaks of the projected 2D chromatin map along the line *L*. We define *H* as the fluorescence difference between the minimum value along *L* and the mean height of the two Gaussians centers (Figure 3d). The value of *H* is significantly greater in menopausal worms than in young worms, suggesting that, indeed, there is less chromatin at the short arms. To further verify these results, we used the Jensen–Shannon divergence to quantify the overlap between the two Gaussian probability distributions in 3D as described (Endres & Schindelin, 2003; Lin, 1991). We found significantly lower levels of overlap between the homologs in menopausal worms than in young worms (Figure 3e), supporting the finding that homologs in menopausal worms are more spatially separated than in younger worms.

Interestingly, when we imaged oocytes in aged COSA‐1::GFP worms, we noticed that 82.5% of the oocytes contained bivalents with double COSA‐1 foci at the chiasmata region (Figure 3f). Together with the DAPI staining, this observation supports the hypothesis that weakening of the sister chromatid cohesion around the chiasmata reduces the binding of the homologs in older oocytes, thus increasing their spatial separation.

### 2.7 Lower quality oocytes are present in aged germline

**FIGURE 4:**
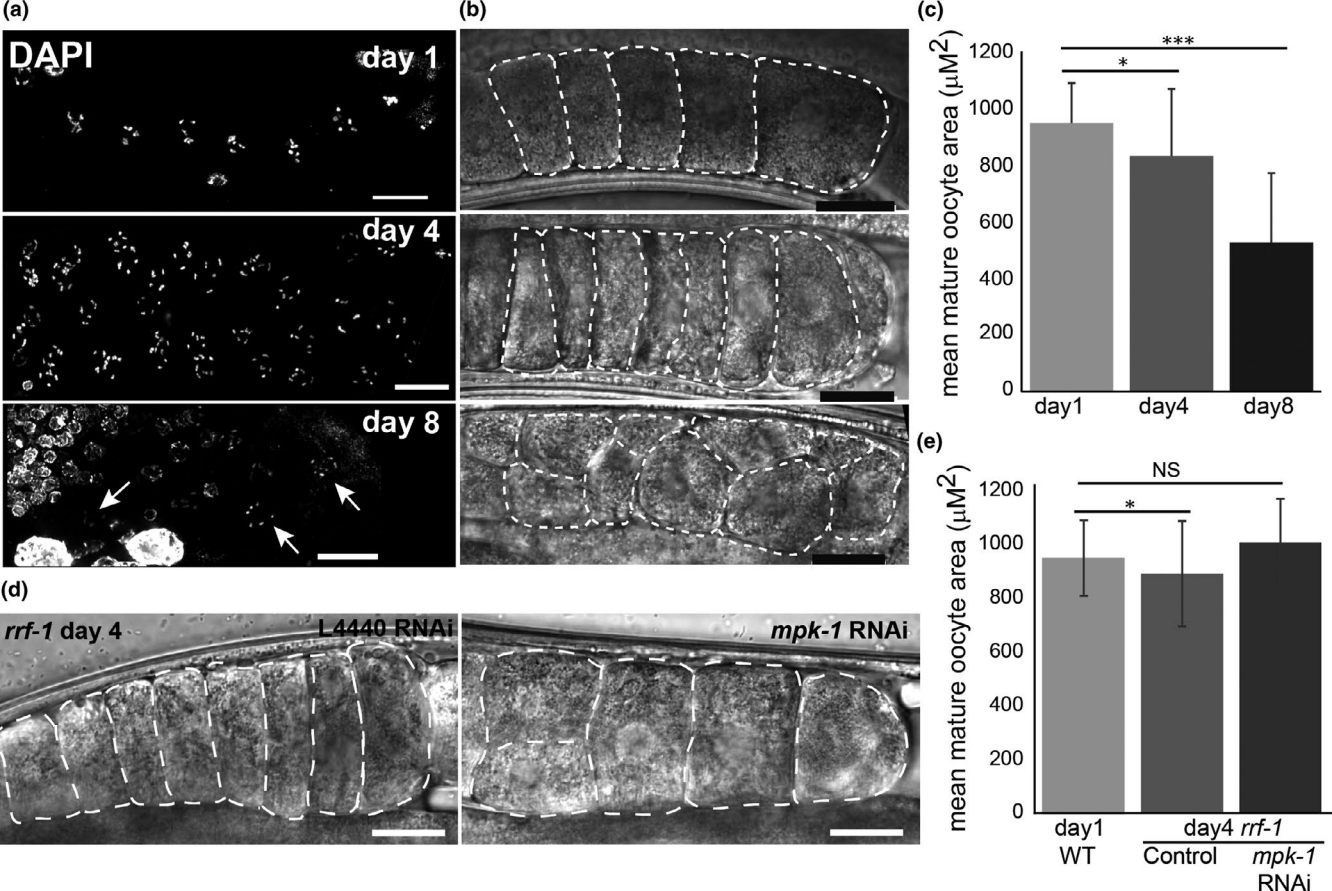
Oocyte get smaller with age. (a and b) Proximal end of gonad arms at the indicated ages (a) stained with DAPI and imaged with fluorescence microscopy and (b) imaged with DIC. Arrows indicate pairs of diakinesis nuclei in the same row. White dashed lines indicate the circumference of the mid plane of oocytes. Scale bar =20 µM. (c) Average area of −1 oocytes at the different ages. *n*=20 oocytes for day 1, *n* = 30 for day 4, and *n* = 11 for day 8. (d) Proximal end of gonad arms of *rrf*‐*1 *day 4 gonads of control and *mpk*‐*1* RNAi worms. White dashed lines indicate the circumference of the mid plane of oocytes. Scale bar =20 µM. (e) Average area of −1 oocytes. *N* ≥ 13 gonads. Mann–Whitney *p* value: NS—not significant, *<0.05, and ***<0.001

In young adult worms, the diakinesis oocytes are stacked sequentially one after the other at the proximal end of the gonad. In aged worms, we found smaller oocytes which were aligned in multiple rows (Figure 4a). Differential interference contrast (DIC) imaging confirmed that oocytes from aged worms were significantly smaller than those from young worms (Figure 4b). Together with the presence of small oocytes, we also detected endomitotic nuclei starting at day 4. The number of endomitotic nuclei increased dramatically with age (32% at day 4, *n* = 18 gonads, 83% at day 8, *n* = 32 and 96% at day 10, *n* = 28; Figure 1c,e). Endomitotic nuclei are oocytes that have bypassed the prophase I diakinesis arrest but that have failed to fully complete anaphase I and likely undergo endoreduplication instead of mitosis (McGee et al., 2012). Collectively, these results suggest that the arrest of oocytes in *C*. *elegans* can lead to various aberrations including lower quality oocytes, defective G2/M arrest, and reduced interhomolog cohesion.

### 2.8 MAPK signaling dynamics changes in aged gonads

**FIGURE 5:**
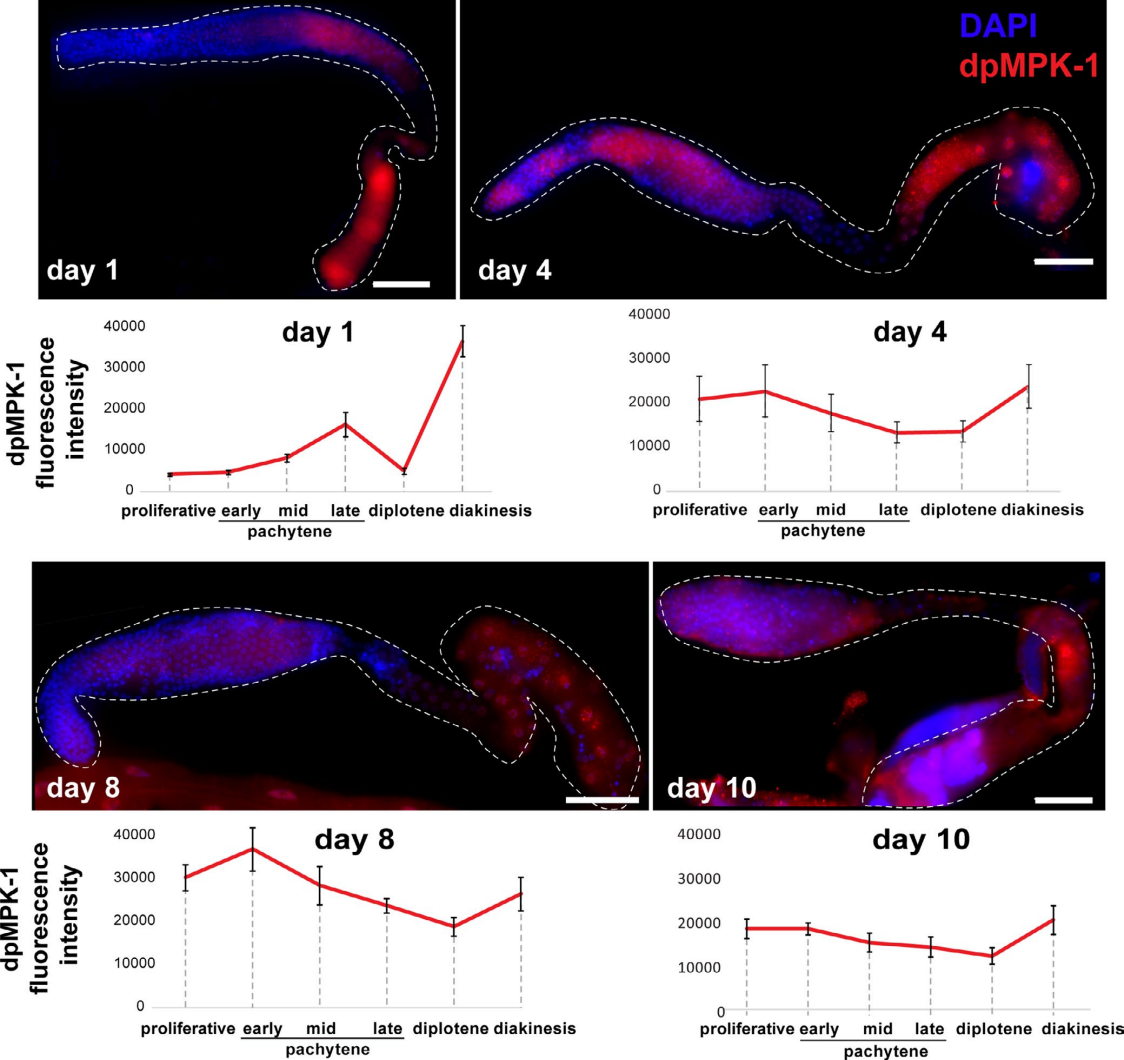
MPK‐1 activation in the gonad changes during aging. Images of whole mount gonads from worms of the indicated ages stained for dpMPK‐1 (red) and DAPI (blue). Average levels of dpMPK‐1 signal are indicated below the images. *n* = 8 gonads for day 1, *n* = 8 for day 4, *n* = 5 for day 8, and *n* = 10 for day 10

We previously reported that a mutation in the *ogr*‐*2* gene results in aberrant meiotic progression, increased apoptosis, and development of small oocytes and endomitotic nuclei (Achache et al., 2019). These phenotypes are extremely similar to those observed in *lip*‐*1* mutants (Achache et al., 2019; Hajnal & Berset, 2002; Lee et al., 2006; Lin & Reinke, 2008). Mutations in either *ogr*‐*2* or *lip*‐*1* genes lead to increased activation of MPK‐1 in several regions of the gonads including mature oocytes at both day 1 and day 4 (Figure S4) (Achache et al., 2019). In addition, mutation in *ogr*‐*2* causes a significant reduction in *lip*‐*1* expression. Therefore, *ogr*‐*2* and *lip*‐*1* were suggested to act via the same pathway to negatively regulate MPK‐1 activation (Achache et al., 2019). The use of the *ogr*‐*2* mutant strain is advantageous in germline studies, because unlike *lip*‐*1*, its expression is limited to this tissue and no somatic defects are observed (Achache et al., 2019). The similarities in the phenotypes of old (8 days) wild‐type worms (Figure 1c) and *lip*‐*1* and *org*‐*2* raise the hypothesis that MPK‐1 activation changes with maternal age. Therefore, we stained the gonads of days 1, 4, 8, and 10 worms with an antibody directed against the phosphorylated (activated) form of MPK‐1 (dpMPK‐1). As was previously demonstrated in young adult worms (Arur et al., 2011; Church et al., 1995; Kritikou et al., 2006; Lackner & Kim, 1998; Lee et al., 2007; Narbonne et al., 2017; Yin et al., 2016;), MPK‐1 activation is restricted to two main regions of the gonad, the mid‐ to late pachytene and the late diakinesis stages (Figure 5). We observed that as the worm ages, MPK‐1 becomes ectopically activated in other regions of the gonad such as the proliferative zone, the early pachytene, and the diplotene stages comparably to young *lip*‐*1* and *ogr*‐*2* adult worms (Figure 5) (Achache et al., 2019). In these strains, the aberrant activation at diplotene led to increased apoptosis (Achache et al., 2019; Perrin et al., 2013; Rutkowski et al., 2011). Importantly, unlike on day 1, at days 4, 8, and 10, we did not detect a peak in dpMPK‐1 levels at late diakinesis (Figure 5). This is probably due to the depletion of sperm known to activate MPK‐1 in diakinesis through the major sperm protein MSP (Han et al., 2010; Miller et al., 2001). Our results indicate that, like oogenesis progression, germline aging is linked to a change in MPK‐1 activation in the gonad.

### 2.9 Aged oocyte quality can be controlled by attenuation of MAPK signaling

**FIGURE 6:**
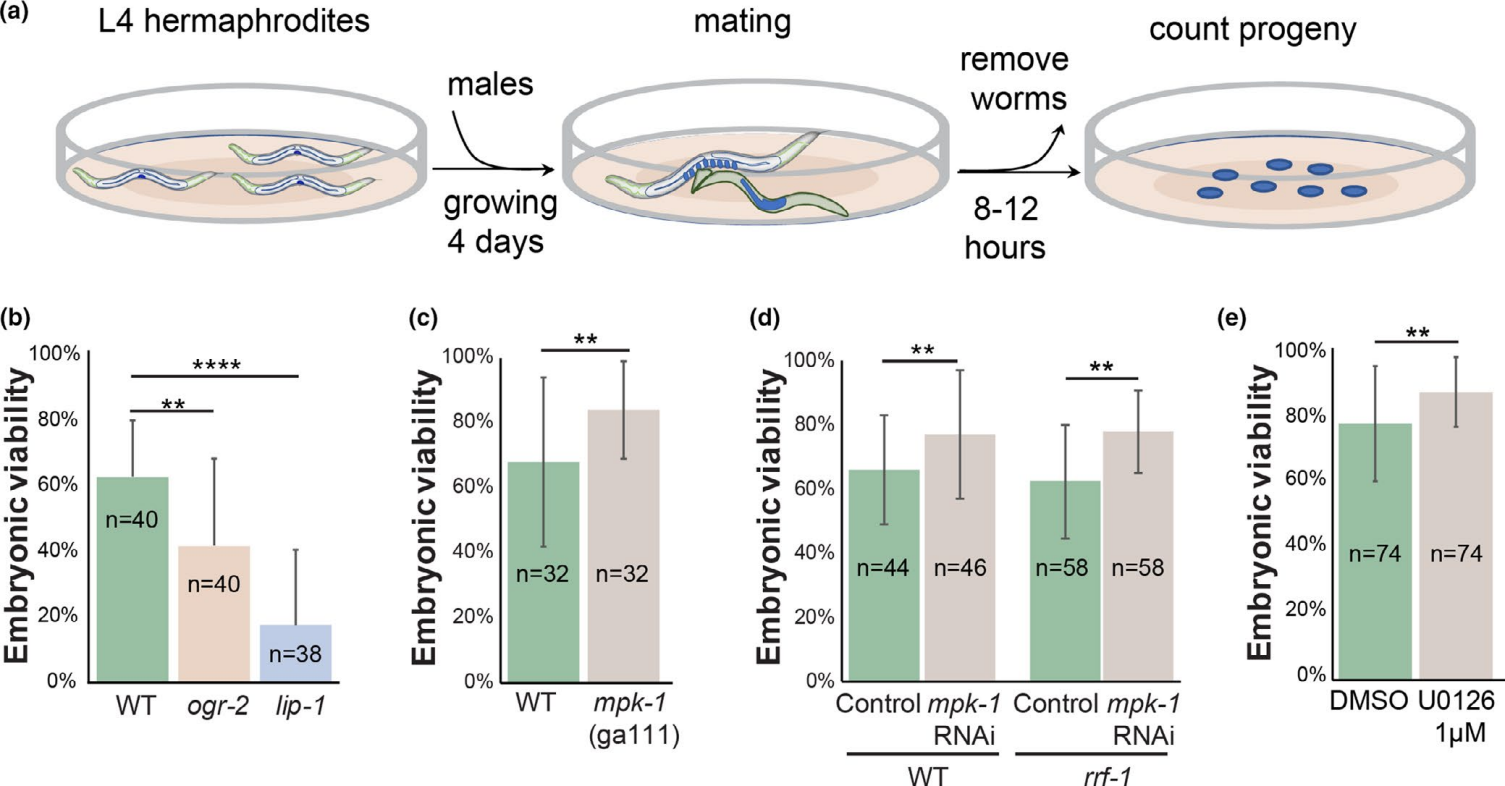
Viability of aged oocytes can be attenuated by MPK‐1 activation. (a) Illustration of the experimental design. (b–e) Average embryonic viability of the first ~10 fertilized oocytes after the arrest in (b). mutants that lead to over activation of MPK‐1 in oocytes, (c) in wild‐type and *mpk*‐*1(ga111)* at semi‐permissive (20 °C) temperature, (d). in wild‐type and *rrf*‐*1* worms depleted of *mpk*‐*1* using RNAi, and (e). in worms that were kept in the presence an MPK‐1 inhibitor during the arrest. Mann–Whitney *p* value: **<0.01 and ****<0.01. n values are indicated on the columns

Our findings that wild‐type oocytes of aged worms have similar morphology to young oocytes with high levels of MPK‐1 activation suggest that MAPK signaling influences oocyte quality throughout aging. To test this hypothesis, we compared the viability of aged‐fertilized oocytes with high and low levels of MAPK activation. This assay was established previously to evaluate oocyte quality (Andux & Ellis, 2008). For higher levels of MPK‐1 activation, we used *lip*‐*1* and *ogr*‐*2* which have increased staining of dpMPK‐1 in mature oocytes (Figure S4 and (Achache et al., 2019)). At 4 days post‐L4, wild‐type, *lip*‐*1*, and *ogr*‐*2* worms were mated with young adult wild‐type males. In these worms, the self‐sperm was depleted approximately 24 hours before mating, so the oocytes were arrested for about one day. Between 8 and 12 hours after the introduction of males, adult worms (hermaphrodites and males) were removed, and the numbers of fertilized embryos were counted (Figure 6a). This time window was chosen because we aimed to evaluate the quality of the embryos that originated from the stacked and aged oocytes only. After this window, the embryos laid could be meiocytes at the pachytene stage during the arrest. The hatched embryos were scored 24 and 48 hours after the removal of adult worms to assess embryonic viability. We found that the embryonic viability in mature wild‐type mated worms was 67±19% (*n* = 40 mated worms). In contrast, the embryonic viability was significantly lower in mutants with higher MPK‐1 activation: 45±28% (*n* = 40) for *ogr*‐*2* and 23%±28% (*n* = 38) for *lip*‐*1* (Figure 6b). The difference between the two mutants could result either from different level of MPK‐1 activation (Figure S4) or from somatic effects that exist in *lip*‐*1* mutants but not in *ogr*‐*2* mutants. The higher MPK‐1 overactivation observed in *lip*‐*1* vs *ogr*‐*2*, that corresponds to the magnitude of the reduction in embryonic viability, supports the former possibility. We conclude that the quality of *lip*‐*1* and *ogr*‐*2* arrested oocytes, where MPK‐1 is overactivated, is lower than wild‐type oocytes.

Our hypothesis that the quality of arrested oocytes is determined by MPK‐1 activation further predicts that reducing the levels of MPK‐1 in the gonads will lead to an improvement in oocyte quality. MPK‐1 mutants either are non‐viable or lead to oogenesis arrest at late pachytene (Church et al., 1995), precluding mating experiments with no MPK‐1 activity. We therefore used the temperature sensitive allele *mpk*‐*1(ga111)*. Using a similar mating protocol, we found that in semi‐restrictive conditions, the viability of embryos laid by *mpk*‐*1(ga111)* aged worms was significantly higher than wild type (Figure 6c, 84±15% vs. 68±26%, respectively, *n* = 32). To verify this result, we used RNA silencing to partially reduce the levels of *mpk*‐*1* in the gonads. We found that the embryonic viability was again significantly increased in oocytes with reduced *mpk*‐*1* expression as compared to control RNAi (77±20%,*n* = 46 vs. 66±17%, *n* = 44, respectively, Figure 6d). To specifically reduce the germline expression of *mpk*‐*1*, we used the *rrf*‐*1* strain in which somatic RNAi is reduced or abolished (Sijen et al., 2001), and found similar effects (78±13% vs. 62±18%, for *mpk*‐*1* RNAi vs control, respectively, *n* = 58, Figure 6d). Moreover, DIC imaging showed that mature *rrf*‐*1* oocytes from day 4 *mpk*‐*1* RNAi worms were significantly larger than those from control worms and even reached the size of young wild‐type oocytes (Figure 4d,e). Together these results suggest that reducing MAPK signaling in arrested oocytes increases their quality and ability to successfully complete meiotic divisions or embryogenesis.

To test this, we used the specific MAPK inhibitor U0126, which was shown to reduce MPK‐1 activation in worm gonads (Morgan et al., 2010; Okuyama et al., 2010). Following self‐sperm depletion, mature worms were moved to media containing the U0126 inhibitor. After 24 hours, the worms were returned to normal NGM plates without the inhibitor, and the quality of the arrested oocytes was assessed by measuring the embryonic viability of the first embryos that were laid after mating. We found that the quality of oocytes from worms exposed to the inhibitor was significantly increased compared to control worms (88±10% vs. 77±17%, *n* = 74, for MPK‐1 inhibitor vs DMSO, respectively, Figure 6e). These results suggest that oocyte quality can be extended by reducing the MAPK signaling, even after oocytes are formed.

Our results support the possibility that oocyte quality in aged worms can be attenuated by MPK‐1 activation. Can other germline aging effects be controlled by MPK‐1? To answer this question, we chose to test DNA double‐strand break repair which has not been previously reported to depend on MPK‐1 activation. We found that when *mpk*‐*1* expression was depleted by RNAi for 24 h in worms that were aged for four days in normal media, RAD‐51 foci were significantly increased in early and mid‐pachytene compared to control, and the values were closer to those observed in young worms (Figure 2f). We conclude that depleting *mpk*‐*1* improves oocyte quality in aging worms and may also delay other aging effects.

### 2.10 Discussion

The classical model of aging defines it as the collective physiological processes that gradually decline, fail, and eventually lead to health deterioration and death overtime (Lopez‐Otin et al., 2013). The importance of focusing on oocyte aging, and the ways it can be delayed, is highlighted by the fact that oogenesis is one of the first processes to fail in humans and worms. Through careful analysis of oogonial processes along four critical points in the reproductive aging of *C*. *elegans*, we found an inherent signaling pathway that regulates the quality of aging oocytes. Our analyses indicate that several processes deteriorate with age. Indeed, we observed that the number of germ cells decreases, nuclei with a crescent shape morphology tend to disappear, the distance between the homologous chromosomes in mature oocytes increases, and there is a reduction in germ cell proliferation, crossover designation, and RAD‐51 foci. In old worms, the oocytes were smaller, and diakinetic nuclei were observed distally. In contrast, there were no differences in either synapsis or chiasmata formation between young and old worms.

An interesting heterogeneity in the organization of the nuclei was observed in the gonads starting at day 8, where instead of reduced number of nuclei, a sub‐population had an over proliferation of cells. In these gonads, we observed a loss in the spatial temporal pattern of the nuclei, and late meiocytes were found distally and vice versa. Increased heterogeneity has been previously observed in other aging processes in *C*. *elegans* (reviewed in (Pincus & Slack, 2010)). One possible explanation for this heterogeneity could be the order the worms were hatched, a factor which has previously been shown to control developmental heterogeneity in worms (Perez et al., 2017). On the cellular level, this disorganization of the gonad could stem from a time restricted loss of control on MAPK in specific nuclei, which can lead to accelerated development of early meiocytes (Yin et al., 2016).

Some of our results can be explained by the halt in ovulation. This halt, which is mediated by the lack of sperm (McCarter et al., 1999), leads to oocyte stacking, and germ cell proliferation reduction, which together with ongoing apoptosis, leads to reduction in germ cell number. The disappearance of LZ nuclei can be the result of developmental progression of early meiocytes nuclei into pachytene, beyond the RAD‐51 removal stage, without spatial movement, which explains the lower number of RAD‐51 foci we found in aging gonads. The stacked oocytes gradually utilize their yolk and become smaller, whereas sister chromatid cohesion gradually weakens leading to homolog distancing and splayed COSA‐1 signal. We note that both COSA‐1::GFP and CED‐1::GFP worms died before reaching the tenth day post‐L4, yet we did not observe any major differences in the gonad morphology between wild type and those strains during earlier aging time points.

Nevertheless, other results cannot be explained by this simplified ovulation model: the loss of oogenesis progression control observed in the disorganized day 10 worms, the increase in apoptosis in day 4 and appearance of endomitotic nuclei in aging oocytes. All these phenotypes have been connected in the past with aberrant MAPK signaling (Achache et al., 2019; Arur et al., 2011; Cha et al., 2012; Church et al., 1995; Hajnal & Berset, 2002; Kritikou et al., 2006; Lackner & Kim, 1998; Lee et al., 2007; Narbonne et al., 2017; Yin et al., 2016), and indeed, we found a dramatic change in the activation dynamics of MPK‐1 in the gonads of aging worms. Together, this led us to suggest that MAPK signaling is a driver of the changes that occur in the aging germline. Several lines of evidence support this possibility. First, we found a dramatic alteration in the dynamics of MPK‐1 activation during aging. Second, we found changes in proliferation, oogenesis staging, apoptosis, crossover designation, size of oocytes, and endomitosis, all previously shown to be controlled by MAPK (Lee et al., 2007). For example, we previously showed that ectopically high MPK‐1 activation in the LZ region is associated with reduced LZ population (Achache et al., 2019), and Yin et al. showed that changes in local MPK‐1 activation are associated with the appearance of diakinesis nuclei distally (Yin et al., 2016). Depletion of *mpk*‐*1* germline expression during adulthood reverses the number of RAD‐51 to younger values (Figure 2f). Most importantly, here we showed that the period during which oocytes are of high quality can be extended or shortened by reducing or increasing, respectively, the level of MPK‐1 activation using genetic and pharmacological tools. Taken together, we suggest that aging leads to a change in MAPK signaling in the gonad. Phosphorylation of downstream targets collectively leads to the different oogonial alternations. The change in MAPK signaling could be the result of germline intrinsic and/or extrinsic signals that come through the gonad sheath cells and/or as a result of lack of sperm (Govindan et al., 2006, 2009; Li et al., 2012; Miller et al., 2003). In this work, we tested the effect of MAPK on aging oocytes quality and found that reduced signaling improves embryo viability and vice versa. Testing if aging of other oogonial processes can be delayed via the tools presented here will be challenging, given the published works which shows that many processes are dependent on normal MAPK signaling (Achache et al., 2019; Hajnal & Berset, 2002; Lee et al., 2006; Lin & Reinke, 2008). In the future, it will be interesting to find whether indeed other oogonial aging effects can be delayed by reduced MAPK signaling at specific regions.

When compared to previous work, one must keep in mind several differences. First, most previous analyses of oocytes at various aging stages (e.g., (Templeman & Murphy, 2018)) used feminized mutants, whereas we strictly used the N2 wild‐type strain. Thus, oocyte aging in our analyses started at the day 3 of adulthood and not at day 1 as in the feminized strains. This difference may change the effects originating from somatic aging. Second, to reduce the number of days one has to move the worms to fresh plates due to the self‐progeny, provide food, and avoid contamination, we kept the worms at 25 ºC. Maintaining the worms at 25 ºC allowed assessment of oocyte aging at day 4 when oocytes start to stack and arrest in contrast to day 5, at which this occurs in worms maintained at 20 ºC. Our results are in agreement with previous publications which used 20 ºC (de la Guardia et al., 2016; Hughes et al., 2007; Kocsisova et al., 2019; Lim et al., 2008; Pickett et al., 2013) in terms of proliferation and oocyte morphology. Nevertheless, caution should be used when comparing aging dynamics between the two temperatures given other works (Bilgir et al., 2013). In aged worms with mutations in genes involved in IIS and TGF‐β signaling, which were shown to extend the fertility term, oocytes are larger and have younger morphology. They also exhibit higher fertilization potential and increased embryonic viability (Luo et al., 2010). In gonads of these aged worms, there are more apoptotic nuclei, and the reproductive population is larger and contains less morphological abnormalities than in wild‐type worms (Luo et al., 2010; Qin & Hubbard, 2015). Similarly, we found that reducing MAPK signaling improves aged oocyte quality, and they produce embryos with higher viability and more RAD‐51 foci than control. Yet, unlike IIS and TGF‐β which exert their effect on reproductive aging from the soma, we showed that it is MAPK expression in the germline that dictates oocyte aging rate. This raises an interesting possibility; it is possible that the IIS and/or TGF‐β affect germline aging by turning MAPK on and off at specific gonadal regions and at specific aging points to control reproductive aging.

Our work, together with that of others (Gruhn et al., 2019; Hughes et al., 2007; Webster & Schuh, 2017; Zielinska et al., 2019), highlights similarities in reproductive aging between worms and mammals. In both systems, oocyte quality reduces with age, and the probability that oogenesis will be completed decreases with age. Changes in crossover were linked to age‐related infertility in humans, and it also changes during worm reproductive aging ((Lim et al., 2008) and this work). Importantly, we found indications that aging leads to reduced sister chromatid cohesion in aged oocytes, as was also recently shown for mouse and human oocytes. If indeed the effects of MAPK on oocyte quality and maturation during aging are evolutionarily conserved, it will be critical to identify intrinsic downstream factors in the pathway that control oocyte aging in order to get deeper insights into human reproductive decline.

A landmark paper published by López‐Otín et al. identified major features that constitute the hallmarks of aging (Lopez‐Otin et al., 2013). Although this publication was focused on lifespan and healthspan, it defined the criteria for an aging hallmark: It appears during normal aging, and its increase leads to accelerated aging and its reduction to slower aging. MAPK signaling can be regarded as a hallmark of oocyte aging by these criteria: First, MPK‐1 activation levels in the oocytes change during normal aging. Second, when MPK‐1 activation is increased as in *lip*‐*1* and *ogr*‐*2* oocytes, they age faster; mutant oocytes are smaller and more prone to pass the G2/M arrest, and the resulting embryo is less likely to complete embryogenesis. Most importantly, decreasing MPK‐1 levels, by both RNAi and pharmacological inhibition, increased the size of aging oocytes and improved embryo viability. We conclude that our results indicate that MAPK is a major signaling pathway that attenuates oocyte aging.

## 3 METHODS AND MATERIALS

### 3.1 Strains and alleles

All strains were cultured under standard conditions at 25°C except for mating experiments (*lip*‐*1*, *ogr*‐*2*, RNAi, and ERK inhibitor), which were conducted at 20°C (Brenner, 1974). The N2 Bristol strain was used as the wild‐type background. Worms were grown on NGM plates with *Escherichia coli* OP50 (Brenner, 1974). The following mutations and chromosome rearrangements were used LGI: *rrf*‐*1*(ok589), LGII: *ogr2*(huj1), *meIs8* [pie‐1p::GFP::cosa‐1 + unc‐119(+)], LGIII: *mpk*‐*1(ga111)*, LGIV: *lip1*(zh15), LGV: bcIs39 [Plim‐7::ced‐1::gfp+lin15(+)].

### 3.2 Reproductive span analysis

To verify the effect of aging on the worm fertility, 400 L4 worms were placed on seeded NGM plates and transferred to new plates every 24 h, and their embryos, nonfertilized oocytes, and hatched progeny were counted for 10 days at 25°C.

### 3.3 Gonad nuclei quantification

The numbers of nuclei at each meiotic stage, from the distal tip to the end of pachytene, were counted manually on DAPI‐stained gonads as was previously described (Achache et al., 2019).

### 3.4 Cytological analysis and immunostaining

Immunostaining of dissected gonads was carried out as described (Colaiacovo et al., 2003; Saito et al., 2009). Worms were permeabilized on Superfrost+slides for 2 min with methanol at −20° and fixed for 30 min in 4% paraformaldehyde in PBS. After blocking with 1% BSA in PBS containing 0.1% Tween 20 (PBST) for 1 h at room temperature, slides were incubated with primary antibody for 1 h at room temperature. After incubation with fluorescent secondary antibody 1 h at room temperature, slides were DAPI‐stained for 10 min at 500 ng/ml, destained 1 h in PBST, and mounted with Vectashield (Vector Laboratories). The primary antibodies used were as follows: rabbit α‐LAB‐1 (1:200, (de Carvalho et al., 2008)), rabbit α‐RAD‐51 (1:10,000, SDIX), mouse α‐MAPK‐YT (1:500, M8159; Sigma), rabbit α‐SYP‐2 (1:200, a kind gift from S. Smolikove), rabbit α‐pH3 (D5692, 1:1000; Sigma), and guinea pig α‐HTP‐3 (1:200, (Goodyer et al., 2008)). The secondary antibodies used were Cy2‐donkey anti‐rabbit, Cy3‐donkey anti‐guinea pig, Cy3‐goat anti‐rabbit, and Cy3‐goat anti‐mouse (all used at 1:500 dilution; Jackson ImmunoResearch Laboratories).

### 3.5 Imaging and microscopy

Images were acquired using the Olympus IX83 fluorescence microscope system (Olympus). Optical z‐sections were collected at 0.30/0.60‐µm increments with a Hamamatsu Orca Flash 4.0 v3 and CellSens Dimension imaging software (Olympus). Images were deconvolved using AutoQuant X3 (Media Cybernetics).

### 3.6 Oocyte size measurement

Measurements were performed on whole worms mounted in M9 and visualized using DIC microscopy (Sulston & Horvitz, 1977). Mid‐oocytes plane areas were measured with ImageJ software.

### 3.7 Quantitative analysis of germ cell apoptosis

Germ cell corpses were scored in adult hermaphrodites using CED‐1::GFP as described (Zhou et al., 2001). Worms were transferred onto a drop of M9 on 1.5% agarose pads on slides and visualized. Statistical analyses were performed using the two‐tailed Mann–Whitney *U* test (95% C.I.).

### 3.8 Quantification of immunofluorescence signals

Activated MPK‐1 fluorescence intensity was quantified on raw images taken from whole‐mounted gonads of wild‐type worms at the different aging phases stained with an anti‐dpMPK‐1 antibody using the same experimental conditions and identical acquisition parameters. ImageJ software was used to measure the fluorescence intensity level throughout the entire length of the gonad.

### 3.9 Time‐course analysis for RAD‐51 foci

The average RAD‐51 foci number per nucleus was scored in each meiotic stage of the germline. Statistical comparisons between the different aging stages were performed using the two‐tailed Mann–Whitney *U* test (95% C.I.).

### 3.10 Quantification of COSA‐1 foci

For quantification of GFP::COSA‐1 foci, nuclei that were in the last four‐to‐five rows of late pachytene and were completely contained within the image stack were analyzed. Foci were quantified manually from deconvolved 3D stacks.

### 3.11 Quantification of the distance between the homologs

DAPI‐stained images of the bivalents were recorded in z‐stacks with vertical separation of Δ*z* 0.3 µm and horizontal pixel resolution of Δx Δy 0.064 µm. To simplify the analysis, we selected bivalents in which the homolog interface (short arms) was parallel to the z axis (Figure 4a). 3D fluorescence data were represented as a 3D matrix such that each voxel *i*, j, k has a measured fluorescence value *F*, i, j. First, we performed clustering‐based segmentation in 3D to isolate the relevant bivalents.

We then fitted the 3D fluorescence data to a Gaussian mixture model (GMM) with two Gaussians. Because GMM fitting operates on a point cloud rather than on a scalar intensity field, we represented the fluorescence matrix as a point cloud, in which the occurrence of each voxel's coordinates is proportional to its fluorescence. Hence, the coordinates of each voxel *i*, j, *k* appeared in the point cloud round 0.1 ⋅ *F i*, j times. The value of 0.1 was chosen to reduce the point cloud's size and speed up computation time; we verified that the results are unchanged when using a value of 1. GMM fitting was performed in MATLAB^TM^ using the fitgmdist function with 20 replicates and 100 iterations per replicate. This procedure resulted in two Gaussians per bivalent, where each Gaussian in described by its mean (point in 3D) and 3 3 standard deviation matrix.

2D fluorescence density maps were obtained by summing all z‐slices in a stack along the z direction. Such projections are shown in Figure 4b, in which fluorescence is represented by both surface height and color code. The mean of each Gaussian was also projected onto the 2D map. The projection of each mean is very close to the two peaks in the 2D map due to the z‐orientation of the bivalents in the raw data. The 2D distance *L* between the two bivalents was defined as the 2D distance between the 2D projected positions of the Gaussian means (Figure 4b). The gap between the bivalent was further characterized by defining *H* as the fluorescence difference between the minimum value along *L* and the mean 2D fluorescence values at the two projected centers of the Gaussians (Figure 4b). The overlap between the bivalents was quantified using the Jensen–Shannon divergence (JSD), which measures the overlap between two probability distributions on a scale between 0 and 1 (Endres & Schindelin, 2003; Lin, 1991). A JSD value of 0 means the two distributions have no overlap, and a JSD value of 1 implies the two distributions are identical. To calculate JSD between the Gaussians fitted to the two bivalents, we first calculated the value of each Gaussian in each voxel and then normalized each Gaussian to 1, to make it a probability distribution. If *P* and *Q* are the values of the two Gaussians in the *n't*h voxel and *M*, *P*, *Q*, then the JSD is given by the sum over all voxels:

$$\text{JSD}\frac{1}{2}‐p\log\frac{P}{M}Q\log\frac{Q}{M}$$

### 3.12 Male generation

Young wild‐type adult males were generated by crossing wild‐type L4 hermaphrodites with wild‐type males and growing for 3 days at 20°C.

### 3.13 RNAi

Feeding RNAi experiments were performed at 20°C as described (Govindan et al., 2006, 2009). The control experiment was performed by feeding HT115 bacteria carrying the empty pL4440 vector. A feeding vector from the *C*. *elegans* RNAi collection (Source Biosciences) was used to deplete *mpk*‐*1*. To study the germline specific functions of MPK‐1 in *C*. *elegans*, we used mutants of *rrf*‐*1* strain, which encodes an RNA‐directed RNA polymerase, to allow RNAi to be effective mostly in the germline (Sijen et al. 2001). Note that a previous report has shown that in some cases, the RNAi in this strain also occurs in somatic tissues (Kumsta & Hansen, 2012).

### 3.14 Oocyte quality experiments

To test aging oocyte quality in mutant strains that have either increased or reduced MPK‐1 activation (*ogr*‐*2*, *lip*‐*1* and ts allele of *mpk*‐*1* (ga111)), we used mating experiments. L4 stage worms were grown on seeded NGM plates for 4 days at 20 ºC. For control, we used the N2 strain. Worms were transferred to new plates every 24 h to remove progeny. After 4 days, they have depleted their stock of sperm. At this point of time, each single hermaphrodite was transferred to a mating plate and mated with five wild‐type males for 8–12 hours. This period corresponds to the time that takes the worms to lay between 10 and 15 eggs originating from stacked oocytes. Hermaphrodites and males were then removed from the plate, and the embryos were counted immediately. Each plate was scored for a second time after 24 hours. Embryos that had not hatched were marked as dead.

To test aging oocyte quality following RNAi depletion, day 3 post‐L4, wild‐type or *rff*‐*1* adult worms grown on normal NGM were placed on either *mpk*‐*1* RNAi or control RNAi plates for 24 hours. They were then transferred back to NGM mating plates and mated as described above. Testing aging oocyte quality with MAPK inhibitor. U0126 (1,4‐diamino‐2,3‐dicyano‐1,4‐bis[2‐aminophenylthio] butadiene monoethanolate) was purchased from Sigma. Worms were aged for 3 days on normal NGM plates and then transferred for 24 h to liquid or solid media containing either DMSO or 1 µM of U0126. Mating and embryo viability scoring was performed as described above.

## CONFLICT OF INTEREST

The authors declare that they have no conflict of interest.

## AUTHORS’ CONTRIBUTIONS

RF and HA performed experiments and analyzed data. TB and NL devised and performed computational and biophysical analysis. HA and YBT designed experiments and wrote the manuscript.

## Supporting information

Fig S1

Fig S2

Fig S3

Fig S4

## Data Availability

Strains and plasmids are available upon request.
