# Supplementary figures and images for: Oocyte aging is controlled by mitogen‐activated protein kinase signaling
Source: Aging Cell. 2021 Jun 1;20(6):e13386. doi: 10.1111/acel.13386 (PMC8208789; doi:10.1111/acel.13386)

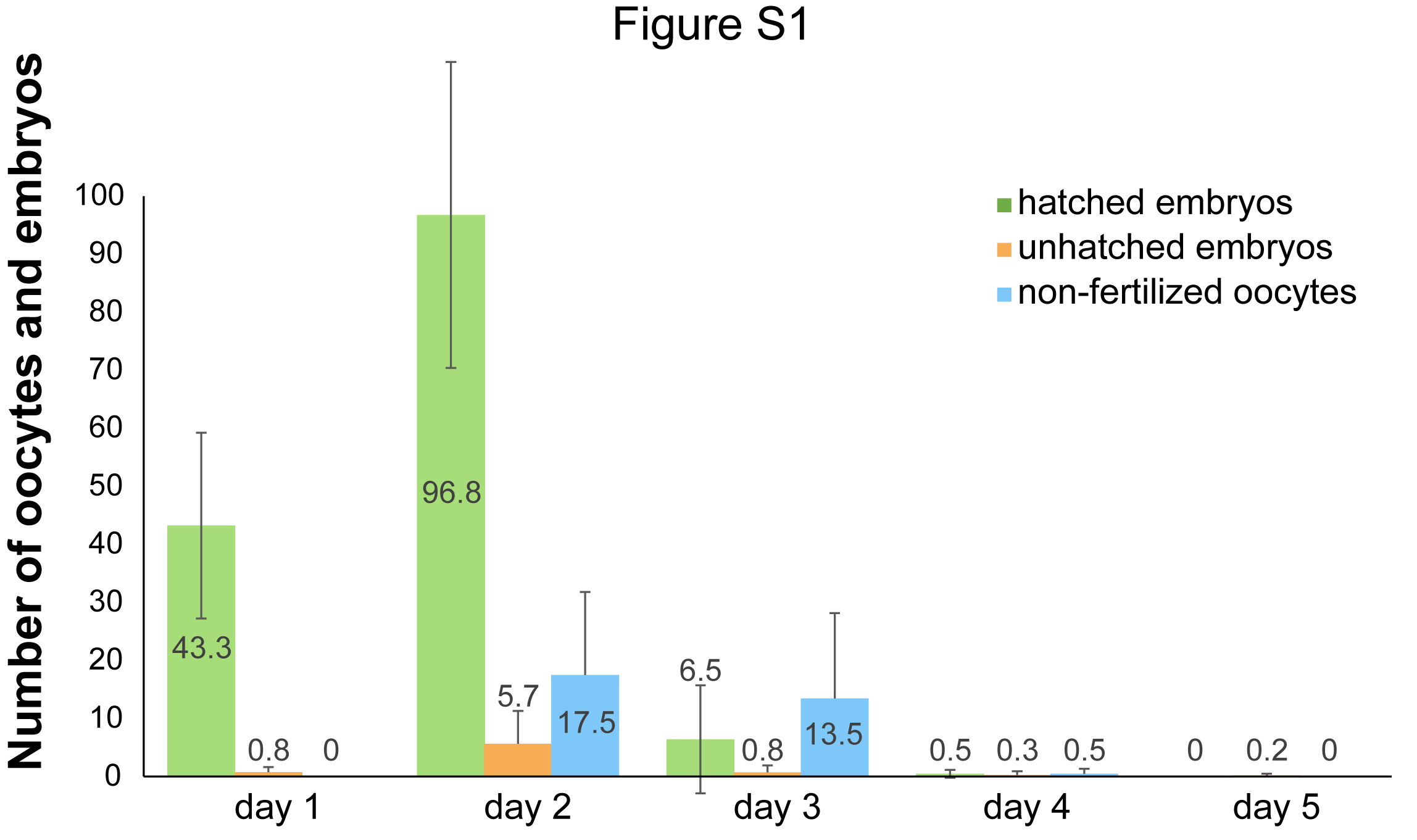

Supplement: Supplementary file 1 — Fig S1 [file ACEL-20-e13386-s002.tif]

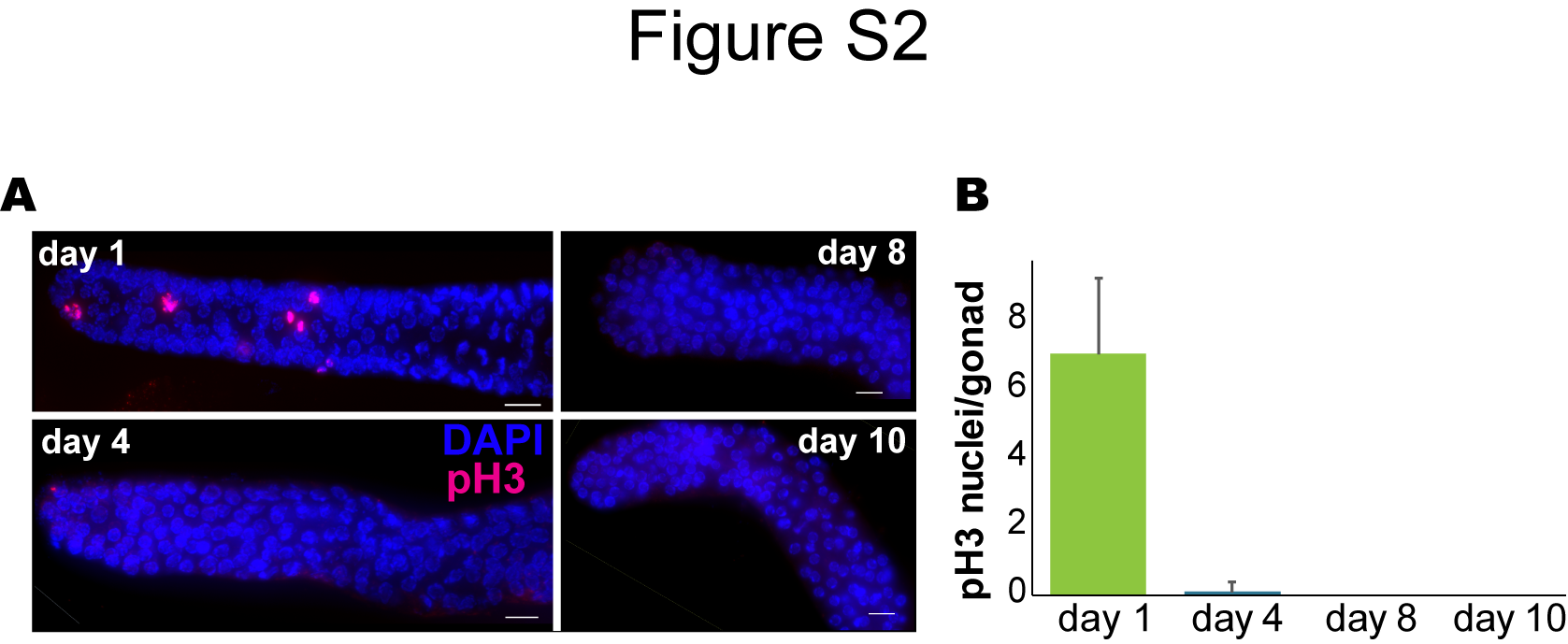

Supplement: Supplementary file 2 — Fig S2 [file ACEL-20-e13386-s003.tif]

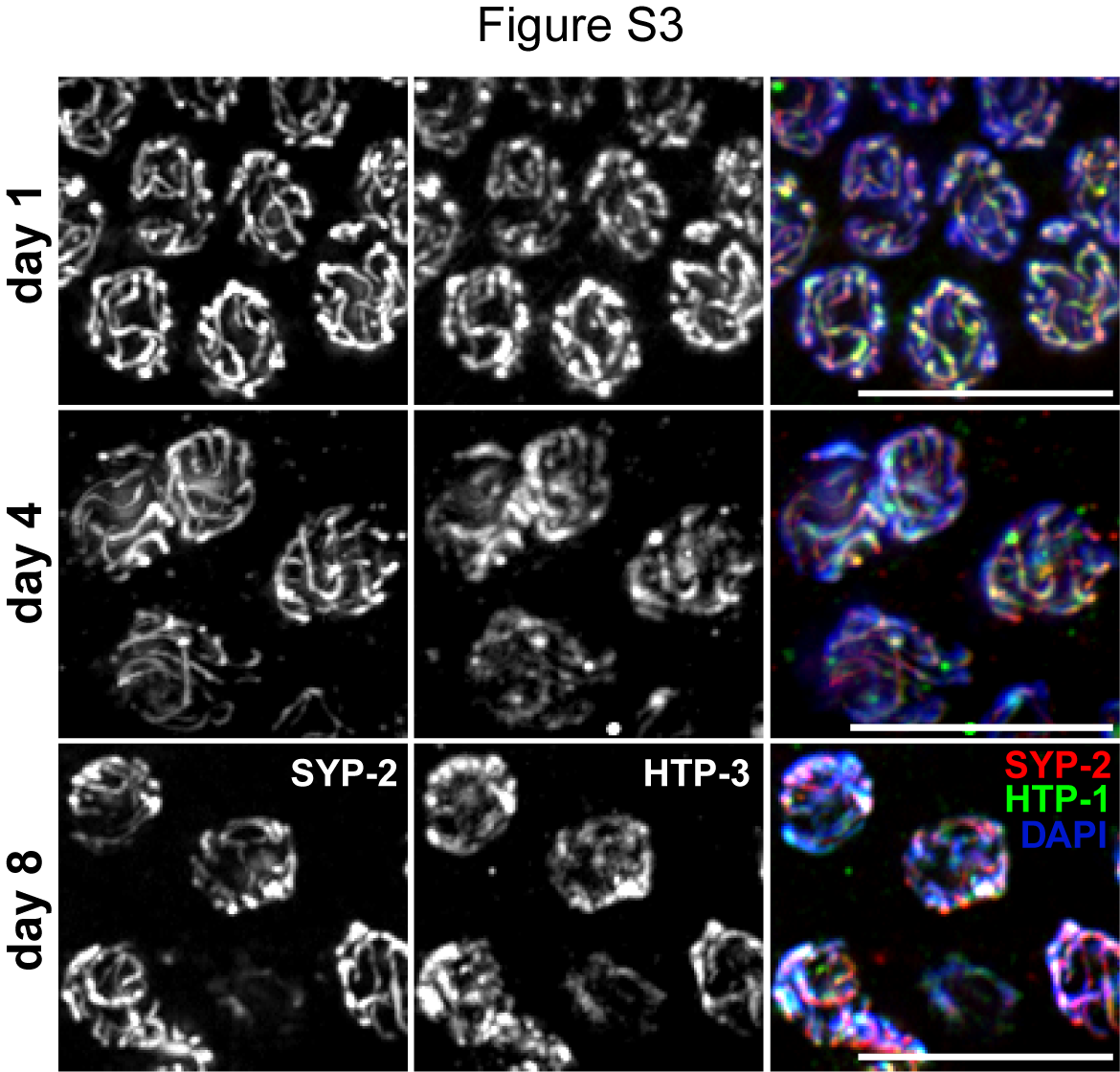

Supplement: Supplementary file 3 — Fig S3 [file ACEL-20-e13386-s001.tif]

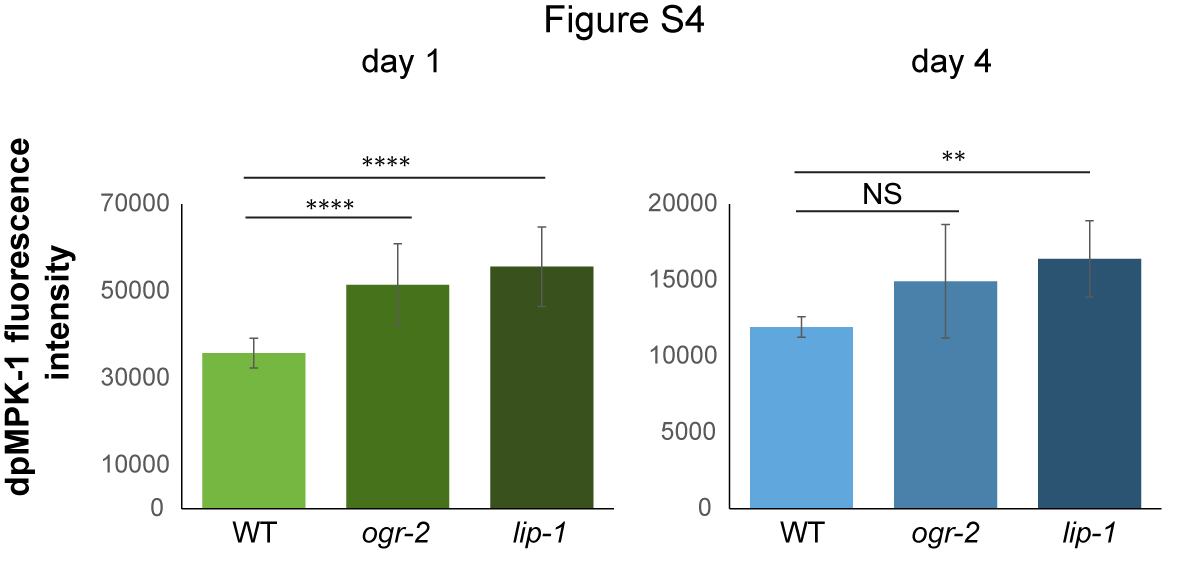

Supplement: Supplementary file 4 — Fig S4 [file ACEL-20-e13386-s004.tif]
